# Viral Hepatitis as a Public Health Concern: A Narrative Review About the Current Scenario and the Way Forward

**DOI:** 10.7759/cureus.21907

**Published:** 2022-02-04

**Authors:** Ajeet S Bhadoria, Anushikha Dhankhar, Giten Khwairakpam, Gagandeep Singh Grover, Vineet Kumar Pathak, Pragya Pandey, Rohit Gupta

**Affiliations:** 1 Community and Family Medicine, All India Institute of Medical Sciences, Rishikesh, Rishikesh, IND; 2 Public Health Sciences, Treat Asia/amfAR, Bangkok, THA; 3 Public Health Sciences, Department of Health & Family Welfare, Chandigarh, IND; 4 Community and Family Medicine, All India Institute of Medical Sciences, Raipur, Raipur, IND; 5 Gastroenterology and Hepatology, All India Institute of Medical Sciences, Rishikesh, Rishikesh, IND

**Keywords:** india, guidelines, epidemiology, viral hepatitis b, viral hepatitis a

## Abstract

Viral hepatitis is one of the emerging public health problems, which urgently needs special attention. The disease has a varied presentation at the time of diagnosis, and it can progress from an accidental finding to life-threatening conditions like liver cirrhosis. It belongs to the rare group of diseases that can cause chronic inflammation inside the body, and it can have a delayed presentation. It contributes substantially to the global burden on healthcare. In terms of mortality, the burden due to viral hepatitis is similar to that of HIV and tuberculosis. It is among the major global public health challenges along with other communicable diseases, such as HIV, malaria, and tuberculosis; the major difference is that there are very limited preventive models in place for viral hepatitis, especially in developing countries like India. With limited resources for diagnosis and treatment, varied levels of presentation, and a rapidly increasing burden, it can become the next silent pandemic. In the current review, the authors aimed to compile the available global strategies for combating hepatitis, protocols available for disease surveillance, and the salient points from the national program for hepatitis control in India [National Viral Hepatitis Control Program (NVHCP)], and propose some recommendations. Ensuring a health facility equipped with a rapid diagnostic kit for screening, proper lab for the confirmation, robust Health Management Information System (HMIS) portal for the data management, and organizing regular workshops for physicians and lab technicians are some of the recommendations that we put forward.

## Introduction and background

Global burden: viral hepatitis as a public health concern

Viral hepatitis refers to a pathologic condition wherein an infection due to hepatitis viruses causes inflammation of the liver [1]. It contributes substantially to the global burden on healthcare, with 248 million people infected with hepatitis B and 71 million infected with hepatitis C worldwide [2]. It is among the major global public health challenges besides other communicable diseases, such as HIV, malaria, and tuberculosis [3]. According to the World Health Organization Progress report on HIV, viral hepatitis, and sexually transmitted infections, the condition was responsible for 1.4 million deaths in the year 2016 [4]. Among all deaths attributed to viral hepatitis, 96% were due to hepatitis B virus (HBV) (48%) and HCV (47%) alone [5]. In addition, viral hepatitis is also among the major causes of mortality in people living with HIV (PLHIV). Among PLHIV, the global estimate of the burden of HIV-HCV coinfection and HIV-HBV coinfection is 2.75 million and 2.6 million respectively [6]. The importance given to viral hepatitis prevention can be gleaned from the fact that it has been selected as Target 3 of the 2030 Agenda for Sustainable Development, which emphasizes an urgent need to prevent and control viral hepatitis [7].

Epidemiology of viral hepatitis (Indian scenario)

The burden of viral hepatitis in India is currently wide-ranged, mainly due to the paucity of data. According to the National Health Profile 2019 report, a total of 1,64,826 cases of viral hepatitis were detected in India in the year 2017, out of which 89,780 were men and 74,509 women, with a total of 537 deaths. Also, out of the 1,64,289 cases reported across India in the year 2017, the top 10 states with the highest burden of viral hepatitis were Bihar, Uttar Pradesh, Madhya Pradesh, Punjab, Delhi, Uttarakhand, Haryana, Maharashtra, and Rajasthan [8]. The proportion of hepatitis B surface antigen (HBsAg)-positive individuals in India ranges from 2 to 8%. It is also estimated that 15-25% of HBsAg carriers may later develop cirrhosis and cancer, consequently resulting in premature deaths. It has also been reported that around 40-50% of all hepatocellular carcinoma (HCC) cases are attributable to chronic HBV infection while HCV is responsible for 12-32% of HCC cases [9]. Also, 20-30% of all cirrhosis cases are due to chronic HBV infection while 12-20% are due to chronic HCV infection [10].

Objective

The present article aims to assess the current status of viral hepatitis national programs and strategies implemented for its prevention, control, and management. The authors aim to compile the available global strategies for hepatitis, protocols available for disease surveillance, and the salient points from the national program for hepatitis control in India [National Viral Hepatitis Control Program (NVHCP)] and offer some recommendations.

## Review

Global strategies: guidelines on hepatitis B and C testing, 2017

Global Health Sector Strategy (GHSS) on Viral Hepatitis, 2016-2021 is the first GHSS on viral hepatitis, a mission that is expected to contribute to the achievement of the 2030 Agenda for Sustainable Development. It covers the first six years of the post-2015 health agenda, 2016-2021, building on the Prevention and Control of Viral Hepatitis Infection: Framework for Global Action [2] and on two resolutions on viral hepatitis adopted by the World Health Assembly in 2010 and in 2014 [11]. The strategy addresses all five hepatitis viruses (hepatitis A, B, C, D, and E), with a particular focus on hepatitis B and C, owing to the relatively severe public health burden they represent. This strategy proposes eliminating viral hepatitis as a public health threat by 2030. The success of this strategy requires the diagnosis of 90% of infected individuals and treatment of 80% of the diagnosed cases with the aim of a 65% reduction in mortality. This strategy was last updated in 2016 and required changes regarding the time and course of treatment owing to three key developments, viz the evolution of direct-acting antiviral (DAA) regimens; a reduction in the need for conducting genotyping after the approval of DAA medicines that are pan-genotypic in nature; rapid rolling out of treatment in low- and middle-income countries due to substantial cost reduction of DAAs. These guidelines entail evidence-based recommendations for program managers and healthcare providers for treating persons with chronic HCV infection. Guidelines for the care and treatment were updated on the principle of "screening, care, and treatment", which were issued by the WHO Guidelines Review Committee in 2017 [12]. Low- and middle-income countries have been reporting the majority of cases of HBV and HCV infections. However, the burden is more pronounced among specific population groups, such as persons who inject drugs (PWID) and individuals from certain indigenous communities.

Protocol for the surveillance of the fraction of cirrhosis and hepatocellular carcinoma attributable to viral hepatitis in clinical centers of excellence, 2018

WHO recommends viral hepatitis surveillance that includes incidence of acute hepatitis and HBV and HCV prevalence, and mortality resulting from sequelae of liver disease, such as cirrhosis and HCC. Mortality reduction among HBV and HCV cases is used in the GHSS as one of the criteria for defining the goal of eliminating viral hepatitis as a public health threat by 2030 [10]. Therefore, laying down approaches to measure HBV- and HCV-related mortality are necessary. Initially, only deaths from acute infections were taken into account for measuring mortality; however, it ignored mortalities associated with chronic liver disease that resulted from hepatitis virus infection, such as cirrhosis and HCC.

Most countries do not have a systematic process to estimate national mortality rates from viral hepatitis, thereby needing to convert ad hoc approaches used in various research studies into routine surveillance systems. For this, deaths resulting from chronic liver disease (including cirrhosis and HCC) and the fraction of disease conditions that can be attributed to various hepatitis viruses can be used to estimate mortality. This protocol describes approaches that can be utilized in sentinel centers (e.g., hepatology or gastroenterology units) to estimate the proportion of individuals with cirrhosis and HCC due to HBV and HCV. This data is crucial to estimate the prevalence of cirrhosis and HCC, individuals in the advanced stage of liver disease, and mortality as a result of cirrhosis or HCC. The cases of cirrhosis and HCC can be followed up to understand the trends in disease development [13].

Hepatitis control through multi-disease testing

Although HIV-associated deaths have been controlled by antiretroviral therapy (ART), morbidity and mortality associated with coinfections like TB, HBV, and HCV pose a threat to investments in treatment. In 2016, approximately 2.75 million HIV patients also suffered from HCV coinfection [14]. Late detection of these coinfections results in infection progressing to an advanced stage by the time the patient reports to a healthcare facility, leading to costly care and management at the patient level. These coinfections also have a high potential for HIV transmission, its progression, and associated mortality. This necessitates the need to scale up screening for HIV and other coinfections in high-risk populations [15,16]. Multi-disease diagnostic platforms can help identify the presence of multiple infections and variations in the pathogen along with associated antimicrobial resistance. This will help streamline and simplify diagnosis and further management of multiple infectious diseases, thereby reducing cost, improving access for patients, and eventually controlling these outbreaks [17].

Elimination of hepatitis C virus infection in children, 2018

Approximately 6-11% of children acquire HCV from their infected mothers [18]. Out of these, only 20% show clearance within two years from birth, while the rest do not and develop chronic HCV infection later in life. As a result, these children are more likely to develop liver disease, which also tends to get severe with age. Adding to the problem is the limited preventive strategies and lack of evidence of their safety. DAAs have revolutionized HCV treatment across the world; however, evidence on their safety during pregnancy is scarce [19].

Standard operating procedures for enhanced reporting of cases of acute hepatitis, 2018

As per WHO, the surveillance of viral hepatitis must cover three key indicators of the disease burden: incidence (new infections of acute hepatitis), prevalence (chronic hepatitis cases), and mortality as a result of sequelae of infection including HCC and liver cirrhosis. To achieve a reduction in the incidence of HBV and HCV, countries need to adopt methods that identify associated risk factors. Although a major proportion of cases (50-70% for HBV and >80% for HCV) are asymptomatic, information on diagnosed symptomatic new infections can prove truly helpful and is the only way to study the change in disease trends among new infections in the community [20].

Progress on viral hepatitis, 2019: global targets, service coverage, and current status

Thanks to the ongoing hepatitis immunization and prevention programs, the incidence of hepatitis infection, especially hepatitis B, has declined. However, the reduction in mortality rates has been negligible, and this requires intervention for improving testing and treatment access. According to the WHO’s Progress Report [4] on HIV, Viral Hepatitis and Sexually Transmitted Diseases, 2019, the targets for 2020 and 2030 for viral hepatitis included a 30% reduction by 2020 and a 90% reduction by 2030 in the number of new chronic viral hepatitis B and C infections. Data suggest that in the year 2017, 1.1 million new cases of chronic hepatitis B were diagnosed; however, for chronic hepatitis C, only baseline data for the year 2015 is available, which reported 1.75 million newly diagnosed cases [21]. For reducing mortality, a target of 10% reduction by 2020 and 65% by 2030 was set for hepatitis B and C. However, only baseline data from the year 2016 is available, showing a total of 1.4 million deaths due to all types of viral hepatitis infections [22].

In order to achieve the 2030 targets for service coverage, the expedition of testing and treatment is necessary. Despite a strong immunization coverage program, the proportion of first doses given at birth is low [8]. Services to prevent mother-to-child transmission of HBV were set to have 50% coverage by 2020 and 90% coverage by 2030. As of 2017, there has been 43% global coverage of the services through a timely hepatitis B vaccination at birth [23]. About 97% of the blood donations were screened for quality assurance (as per baseline data of 2015) against a target of achieving 95% screening by 2020 and 100% by 2030 [2]. In the year 2017, 3.9% injection reuse was reported against a target of 50% injections to be administered following safety-engineering devices by 2020 and 90% by 2030. Harm-reduction services have also been observed to be in urgent need of action with only 33 needle sets provided per injection drug user in 2017 against the target of 200 sterile needle sets per injection drug user by 2020 and 300 by 2030 [24]. Diagnostic and treatment services also need to be made more equitable and accessible to achieve a target of diagnosing 30% of chronic viral hepatitis B and C cases by 2020 and 90% by 2030. However, only 10% (27 million) [25] of people with chronic hepatitis B and 19% (13.1 million) with chronic hepatitis C knew their status as per the 2016 baseline data. Out of these, only 4.5 million (17%) eligible and diagnosed chronic hepatitis B cases had been treated by the year 2016 and only five million diagnosed chronic hepatitis C cases by the year 2017 against a global target of 80% treatment coverage by 2030 [21].

Consolidated strategic information guidelines for viral hepatitis, 2019: planning and tracking progress towards elimination, 2019

Aiming to eliminate viral hepatitis as a public health threat by 2030, the first GHSS on viral hepatitis was endorsed by the World Health Assembly in the year 2016. The GHSS includes the implementation of five core interventions at an acceptable level of service coverage. These five core interventions include immunization, preventing vertical transmission of HBV, safe injection and blood transfusion, harm reduction for high-risk populations (e.g., those using injectable drugs), and testing and treatment. For the purpose of monitoring and evaluation, surveillance and documentation of cases, prevention, testing, and treatment of viral hepatitis have been implemented. The strategy also emphasizes the importance of implementing collaborative efforts in viral hepatitis programs with other different health programs, such as communicable disease control, HIV, TB, cancer care, immunization, and primary care to assist data collection and analysis without the need to set up separate systems [26].

Current status of National Viral Hepatitis Control Program (NVHCP): widening the scope

In India, HBsAg positivity in the general population ranges from 1.1 to 12.2%, with an average prevalence of 3-4%. Anti-HCV antibody prevalence in the general population is estimated to be between 0.09-15% [27]. Population-based syndromic and health facility-based surveillance of viral hepatitis is mandated under the Integrated Disease Surveillance Programme (IDSP). In India, approximately 40 million people are chronically infected with hepatitis B and 6-12 million people with hepatitis C. On the occasion of World Hepatitis Day on 28th July 2018, the Ministry of Health and Family Welfare, Government of India launched the NVHCP [28]. The program covers all types of viral hepatitis infections (from A to E) and comprehensively focuses on all aspects, such as prevention of infection, early identification and treatment, and mapping of treatment outcomes. The program was launched along with the release of its operational guidelines, national laboratory guidelines for viral hepatitis testing, and national guidelines for the diagnosis and management of viral hepatitis.

On 24th February 2019, an advocacy event, "India’s Response to Viral Hepatitis", was held and Technical Guidelines for Diagnosis and Management of Hepatitis B and National Action Plan-Combating Viral Hepatitis in India were released, and the NVHCP website was launched. On World Hepatitis Day, 2019, the inauguration of the model treatment program was conducted at Sion Hospital, Mumbai, and the user manual of the NVHCP-MIS for hepatitis C and a national helpline number for viral hepatitis (1800-11-6666) were launched. Additionally, social media campaigns on prevention, identification of high-risk groups, the risk among pregnant women, as well as treatment-oriented campaigns about free drugs and diagnostics for hepatitis B and C, and laboratory testing and management of viral hepatitis were also created and run.

The program adopted an integrated approach and collaborated with other programs and schemes to provide a promotive, preventive, and curative package of services for individuals suffering from viral hepatitis. Under the ‘Training of Trainers’ initiative of the program, 800 experts were trained on diagnosis and management of viral hepatitis and NVHCP-MIS, while training on viral hepatitis, modes of transmission, and prevention were conducted for community members. Regular review and coordination meetings were also held. In addition, two national-level workshops were held for nodal officers to sensitize them about the services provided under the program. For state principal secretaries, mission directors, and state nodal officers for monitoring the program, video conferences were held twice.

The program has also seen major changes with respect to the existing infrastructure. Currently, model treatment centers have been established in all states and six union territories (UTs). This includes the establishment of 301 treatment centers in 285 districts for service delivery under the program. Nine states have made treatment sites functional in all the districts: Bihar, Haryana, Jharkhand, Kerala, Maharashtra, Mizoram, Nagaland, Punjab, and Rajasthan. Approximately 6.5 lakh serological tests have been done as of now for the diagnosis of viral hepatitis C and 16 lakh tests for hepatitis B, and more than 38,000 patients have been put on treatment for hepatitis C. The infection safety committee has been reconstituted. For enhancing skills and capacity building, the Extension for Community Healthcare Outcomes (ECHO) platform is being utilized for online clinical case discussion and conducting training for healthcare workers.

The main activities of the program involve prevention, diagnosis and treatment, monitoring and evaluation, surveillance and research, and review meetings. The NVHCP is coordinated by the center and state bodies. The National Viral Hepatitis Management Unit (NVHMU) is the center-level body and it controls and evaluates the implementation of programs across the country. The State Viral Hepatitis Management Unit (SVHMU) manages the program at the state level through a nodal officer of the State Health Society. At the district level, the program officer of the District Viral Hepatitis Management Unit (DVHMU) supervises program implementation and manages various activities such as supply chain management, outreach services, and logistics and training. Regarding the allocation of funds, the NVHMU at the center level is responsible for developing a program implementation plan (PIP) for states for achieving annual targets, and supervising, monitoring, and evaluating the overall program. The SVHMU assigns a nodal officer for program implementation at the state and district levels and develops PIP to be discussed and improvised after the approval from NVHMU. The DVHMU deals with ensuring the functionality of labs and treatment centers, identifying potential service delivery sites, training of staff, developing referral linkages, ensuring multi-program linkage, IEC material distribution, and data collection and reporting at the district level.

With the focus on the chronicity of the disease, the first cornerstone key component of the program was prevention through awareness generation, immunization against hepatitis B, and adhering to safe blood and injection practice. Early diagnosis through screening of pregnant women, and community involvement can boost adherence if treatment is placed as a second key component. Under the monitoring and evaluation component, effective linkages to the surveillance system would be established and operational research would be undertaken through the Department of Health Research (DHR) through an online web-based system. The last component as training and capacity building would be a continuous process and will be supported by National Centre for Disease Control (NCDC), the Institute of Liver and Biliary Sciences (ILBS), and state tertiary care institutes and coordinated by NVHCP. It would include the traditional cascade model of training through master trainers and various platforms available for enabling electronic, e-learning, and e-courses.

Challenges

With the program being in its fourth year, a few challenges have been faced in successfully implementing it. The first concern is infrastructure and material management. Proper planning for the estimation of drugs and rapid diagnostic kits is a matter of huge concern for timely procurement and dissemination of materials. Also, the mobilization of human resources for effective service delivery in each district is a big challenge. Optimum procurement of quality testing kits, increasing viral load testing, reporting on the MIS platform, regular monitoring, and review of the program along with supportive supervision at all levels are some focus areas needing an immediate call to action.

Recommendations

Since the program is using preexisting healthcare infrastructure, it should be ensured that the healthcare facility that is designated as a treatment center is provided with rapid diagnostic kits for screening, machinery required for lab investigation, drugs, and a well-developed Health Management Information System (HMIS) portal. Program management units should disburse screening kits in a planned way for maximum utilization; this includes the distribution of kits in a tiered manner by following a top-to-bottom approach. Free-of-cost screening kits should be made available for screening key and bridge populations, as well as antenatal and preoperative screening. The willingness among the population and yield of screening for hepatitis should be studied [29]. There should be a well-established system for notification, confirmatory and auxiliary tests as well as treatment of rapid diagnostic test-positive patients. A decentralized approach could be adopted for better outcomes [30].

Many treatment centers (district hospitals) are not equipped with facilities for basic laboratory investigations to manage viral hepatitis, such as coagulation profile (PT/INR); in some centers, there is no HMIS portal, while in some other centers, even though screening kits and drugs are available, physicians are not aware of the program through which the logistics have been supplied. A well-developed HMIS portal may resolve these issues. Organizations like the Clinton Health Access Initiative (CHAI) and ECHO have shown promising results in the state of Punjab, and other states could also take assistance from these organizations for data management and capacity building [31,32]. The program has the provision for appointing human resources in the form of a medical officer, laboratory technician, data entry operator, and peer supporter. However, at some of the management units and treatment centers, the required staff has not been appointed yet; instead, there is a provision for incentivizing the preexisting healthcare workers. Considering the burden of patients, the feasibility of incentivization is questionable [33]. Moreover, many of these healthcare workers are appointed by the state and report to the chief medical officer at the district level rather than the district nodal officer for NVHCP, which sets two parallel systems in place; commissioning human resources exclusive to run the program could resolve this issue. Strengthening peer support can enable patient engagement with healthcare services, particularly for marginalized populations [34]. Under NVHCP, training workshops for physicians and lab technicians are routinely organized. The scope of such workshops needs to be broadened to include program managers, data entry operators, and peer supporters in order to have well-oriented and accountable staff at every level. Capacity-building should be synchronized with the development of infrastructure and human resources. Judicious nomination of specialty resources should be done for training, with a focus on training internists from centers where infrastructure and human resources have been provided.

There is a necessity for regular monitoring and conducting evaluation meetings and visits, as this can help understand the strengths and gaps in the implementation of the program at the grassroots level. Finally, based on the ground reports, state steering committees should remodel the operational guidelines for each state. It is also recommended to publish these reports to disseminate the best practices and strategies to overcome challenges.

## Conclusions

The burden of viral hepatitis is increasing globally as well as in India. Being among the most populous countries, India contributes significantly to the disease burden. Despite having prevalence rates similar to HIV, tuberculosis, and malaria, political commitment and efforts made in the direction of prevention and control of viral hepatitis need more attention. Although the current scenario with respect to NVHCP has improved over the past few years, infrastructural, resource-related, and service-provision scenarios are still in a budding stage and need an urgent call to action owing to the growing burden of the disease at a faster rate than before. The program has a long way to go; however, with better initiatives at each level of the continuum of hepatitis control and prevention, appropriate action and care can contribute to improving the current trends and can accelerate the progress towards achieving the Sustainable Development Goal 3.3 sooner than expected.
