# Correction: Viral Hepatitis as a Public Health Concern: A Narrative Review About the Current Scenario and the Way Forward

**DOI:** 10.7759/cureus.c64

**Published:** 2022-04-12

**Authors:** Ajeet S Bhadoria, Anushikha Dhankhar, Giten Khwairakpam, Gagandeep Singh Grover, Vineet Kumar Pathak, Pragya Pandey, Rohit Gupta

**Affiliations:** 1 Community and Family Medicine, All India Institute of Medical Sciences, Rishikesh, Rishikesh, IND; 2 Public Health Sciences, Treat Asia/amfAR, Bangkok, THA; 3 Public Health Sciences, Department of Health & Family Welfare, Chandigarh, IND; 4 Community and Family Medicine, All India Institute of Medical Sciences, Raipur, Raipur, IND; 5 Gastroenterology and Hepatology, All India Institute of Medical Sciences, Rishikesh, Rishikesh, IND

The authors contacted Cureus after publication stating that they "forgot to add the name of one of our co-authors at the time of submission of the manuscript" with a request to add the forgotten author. The author, Anushikha Dhankhar, has now been added in the second author position. The authors regret the error.

